# Orange (*Citrus sinensis*) essential oil as feed additive for sheep: nutrient intake and apparent digestibility, nitrogen balance, and rumen fermentation characteristics

**DOI:** 10.1007/s11250-026-04969-z

**Published:** 2026-03-09

**Authors:** Paulo César G. Dias Junior, Ana Carolina S. Vicente, Isabela J. dos Santos, Letícia C. B. Soares, Adrielly L. A. da Silva, Rhaissa G. de Assis, Jamile Haddad Comelli, Daniel M. Polizel, Janaina S. Biava, Diogo F. A. Costa, Alexandre V. Pires, Evandro M. Ferreira

**Affiliations:** 1 Department of Animal Science, “Luiz de Queiroz” College of Agriculture, University of São Paulo, Pádua Dias Avenue, n 11, PO Box 09, Piracicaba, São Paulo, São Paulo 13418-900 Brazil; 2 Department of Veterinary Clinical, School of Veterinary Medicine and Animal Science, São Paulo State University (UNESP), Prof. Doutor Walter Maurício Corrêa Street, s/n, Science, Botucatu, São Paulo, 18618-681 Brazil; 3 Department of Nutrition and Animal Production, FMVZ, University of São Paulo, Duque de Caxias North Avenue, n 225, Pirassununga, São Paulo, 13635-000 Brazil; 4 School of Health, Medical and Applied Science, Institute for Future Farming Systems, CQUniversity, Rockhampton, QLD 4701 Australia

**Keywords:** Additive, Limonene, Metabolism, Sodium monensin, Sheep, Rumen

## Abstract

Feed additives have been used in high-concentrate diets to modulate ruminal fermentation; however, information on the metabolic effects of orange essential oil (OEO) under this dietary condition remains limited. The study aimed to evaluate the effects of increasing levels of orange essential oil as a source of D-limonene on nutrient intake, apparent digestibility, nitrogen balance, and ruminal fermentation characteristics in sheep fed high-concentrate diets, compared with sodium monensin. Ten rumen-cannulated Dorper × Santa Inês wethers were assigned to a 5 × 5 Latin square design and fed diets containing 90% concentrate. Treatments consisted of four OEO inclusion levels (0, 100, 500, and 1000 mg/kg dry matter [DM]) and sodium monensin (25 mg/kg DM; M25). Nutrient intake, apparent digestibility, nitrogen balance, ruminal pH, and short-chain fatty acid (SCFA) concentrations were evaluated. The OEO inclusion did not affect overall nutrient intake (*P* > 0.05). However, intake of dry matter, organic matter, crude protein, neutral detergent fiber, non-fiber carbohydrates (NFC), and total digestible nutrients was higher for OEO treatments compared with M25 (*P* < 0.05). Apparent digestibility of NFC decreased at the highest OEO level (1000 mg/kg DM; *P* < 0.05), although values remained higher than those observed with M25. Digestibility of other nutrients, nitrogen balance, ruminal pH, and SCFA profiles were not affected by OEO inclusion (*P* > 0.05). Under the conditions of this study, orange essential oil did not improve nutrient digestibility, nitrogen balance, or ruminal fermentation in sheep fed high-concentrate diets, although high inclusion levels negatively affected NFC digestibility. These results contribute to understanding the metabolic effects of limonene-rich essential oils as alternatives to ionophores in intensive feeding systems.

**Supplementary Information:**

The online version contains supplementary material available at https://doi.org/10.1007/s11250-026-04969-z.

## Introduction

Sodium monensin is one of the most widely used feed additives in ruminant nutrition due to its effects on ruminal fermentation modulation, nutrient utilization, and feed efficiency (FE) (Goodrich et al. 1984; Russel and Strobel, 1989; Duffield et al. 2012). However, the use of ionophores has increasingly come under scrutiny because they are classified as antibiotics, raising concerns about public perception and potential links to antimicrobial resistance (Abadi et al. 2019; Ben et al. 2019; He et al. 2020). In this context, natural feed additives, particularly essential oils, have been proposed as alternatives to ionophores owing to their antimicrobial properties and potential to modulate ruminal fermentation (Benchaar et al. 2008; Torres et al. 2020, 2021).

Among essential oils, orange essential oil (OEO) stands out for its high availability and elevated concentration of D-limonene, which typically comprises over 90% of its volatile composition (González-Mas et al. 2019). D-Limonene is a monocyclic monoterpene widely distributed in nature and recognized for its antimicrobial activity (Arrieta et al. 2013; Gupta et al. 2021). Previous studies suggest that D-limonene can influence ruminal fermentation by altering the profile of short-chain fatty acids (SCFA) and reducing methane and ammonia production. Nevertheless, most of this evidence stems from in vitro experiments, and the reported effects are highly variable, depending on the inclusion level and diet composition (Crane et al. 1957; Sallam and Abdelgaleil 2010; Rofiq et al. 2021; Dorantes-Iturbide et al. 2022).

In vivo studies with D-limonene-rich essential oils remain limited and inconsistent. Some have reported changes in dry matter intake (DMI), FE, or nitrogen metabolism (Kotsampasi et al. 2018; Dias Junior et al., 2023a, 2023b), while others observed minimal effects on ruminal fermentation characteristics. Notably, most available in vivo studies featured moderate concentrate inclusion in the diet. In diets with high concentrate proportions, where ruminal fermentation dynamics and microbial populations differ substantially, there is a dearth of information on the effects of OEO as a D-limonene source.

Given this background, a clear knowledge gap exists regarding the effects of OEO as a D-limonene source on nutrient intake, apparent digestibility, nitrogen balance, and ruminal fermentation in sheep fed diets with high concentrate inclusion. We hypothesized that increasing levels of OEO would exert antimicrobial effects capable of modulating ruminal fermentation and potentially affecting nutrient digestibility and nitrogen utilization. Thus, the objective of this study was to evaluate the effects of different OEO inclusion levels on nutrient intake, apparent digestibility, nitrogen balance, and ruminal fermentation characteristics in sheep fed diets containing 90% concentrate, and to compare them with those of sodium monensin.

## Materials and methods

The experiment took place at SIPOC (Sheep and Goat Intensive Production System “Ivanete Susin”; 22º 42’24” S and 47º 37’ 53” W), Brazil. All animal-related procedures followed the guidelines for Animal Research Ethics Committee of the University of São Paulo (protocol number 5160100220).

### Animals, design and diets

Ten rumen-cannulated Dorper × Santa Inês wethers [38.8 ± 2.58 kg initial body weight (BW); age = approximately 24 months old] were used in a replicated 5 × 5 Latin square design experiment with 5 periods of 27-d, consisting of a 21-d adaptation followed by 6-d sample collection. Wethers were housed in individual metabolic crates (1.30 × 0.55 m), designed to allow the separation and collection of urine and feces. Crates were kept in a shaded open-sided barn and the rams had *ad libitum* access to water during the experiment.

The experimental diets consisted of five treatments: **OEO0** – (negative control) inclusion of 0 mg/kg DM of OEO (CP Kelco Brasil S/A – Matão, SP, Brazil); **OEO100** - inclusion of 100 mg/kg DM of OEO; **OEO500** - inclusion of 500 mg/kg DM of OEO; **OEO1000** - inclusion of 1000 mg/kg DM of OEO; **M25** - (positive control) inclusion 25 mg/kg DM of sodium monensin (Rumensin 100, Elanco Animal Health, São Paulo, SP, Brazil). The concentrate-to-forage [Coastcross (*Cynodon dactylon*) hay] ratio was 90:10 (DM basis) in all treatments. The experimental diets were formulated to be isoenergetic and isonitrogenous (Table 1) using the Small Ruminant Nutrition System (Cannas et al. 2004).

**Table 1:** Ingredients and chemical composition of the total mixed ration [% of dry matter (DM)]

Item	Treatments¹				
	OEO0	OEO100	OEO500	OEO1000	M25
*Ingredients (%)*					
Coastcross hay (*Cynodon sp*)^2^	10.0	10.0	10.0	10.0	10.0
Ground corn grain^3^	72.0	72.0	72.0	72.0	72.0
Soybean meal	14.0	14.0	14.0	14.0	14.0
Urea	0.4	0.4	0.4	0.4	0.4
Ammonium chloride	0.5	0.5	0.5	0.5	0.5
Limestone	1.4	1.4	1.4	1.4	1.4
Mineral premix^5^	1.7	1.7	1.7	1.7	1.7
Orange essential oil^6^, mg/kg DM	0	100	500	1000	—
Sodium monensin^7^, mg/kg DM	—	—	—	—	25
*Chemical composition*^8^ *(%)*					
Dry matter	85.0 ± 0.52	85.7 ± 0.44	85.6 ± 0.55	85.4 ± 0.70	85.4 ± 0.93
Organic matter	94.5 ± 0.28	94.7 ± 0.13	94.4 ± 0.19	94.4 ± 0.11	94.1 ± 0.36
Crude protein	16.2 ± 0.52	16.3 ± 0.26	16.4 ± 0.55	16.2 ± 0.41	16.2 ± 1.17
Neutral detergent fiber	15.8 ± 1.27	15.8 ± 0.84	15.8 ± 1.02	16.0 ± 1.14	15.8 ± 1.06
Acid detergent fiber	6.3 ± 1.01	6.1 ± 0.74	6.0 ± 0.88	6.0 ± 0.79	6.2 ± 0.77
Ether extract	3.4 ± 0.47	3.4 ± 0.47	3.4 ± 0.45	3.4 ± 0.47	3.4 ± 0.56
Non-fibrous carbohydrate	58.9 ± 0.63	59.1 ± 0.33	58.7 ± 0.54	58.8 ± 0.23	57.7 ± 0.67
ME^9^, Mcal/kg of DM	2.9	2.9	2.9	2.9	2.9

^1^OEO0: inclusion of 0 mg/kg DM of orange essential oil; OEO100: inclusion of 100 mg/kg DM of orange essential oil; OEO500: inclusion of 500 mg/kg DM of orange essential oil; OEO1000: inclusion of 1000 mg/kg DM of orange essential oil; M25: inclusion of 25 mg/kg DM of sodium monensin

^2^Coastcross hay (*n* = 5): DM: 82.9% (± 1.16); OM: 91.3% (± 0.57); CP: 6.5% (± 0.70); NDF: 74.3% (± 2.50); ADF: 38.2% (± 1.45); EE: 1.4% (± 0.25)

^3^Ground corn grain (*n* = 5): DM: 88.2% (± 0.76); OM: 96.6% (± 0.76); CP: 9.2% (± 0.29); NDF: 11.4% (± 0.47); ADF: 2.4% (± 0.36); EE: 3.6% (± 0.23)

^4^Soybean meal (*n* = 5): DM: 87.9% (± 0.30); OM: 93.4% (± 0.10); CP: 45.8% (± 0.65); NDF: 11.5% (± 0.18); ADF: 6.7% (± 0.30); EE: 2.3% (± 0.21)

^5^ Ca: 150 g/kg; P: 40 g/kg; Na: 118 g/kg; Mg: 8 g/kg; S: 19 g/kg; Cu: 300 mg/kg; Mn: 1.250 mg/kg; Zn: 6.480 mg/kg; I: 80 mg/kg; Co: 40 mg/kg; Se: 27 mg/kg; F: 400 mg/kg

^6^Orange essential oil: 95.1% de D-limonene, 3.8% de myrcene e 1.1% de α-pinene (CP Kelco Brasil S/A – Matão, SP, Brazil)

^7^Rumensin 100 (Sodium monensin, Elanco Animal Health, São Paulo, SP, Brazil)

^8^Consider three samples per treatment (*n* = 5)

^9^ME: metabolic energy, estimated using the *Small Ruminant Nutrition System* (Cannas et al. 2004)

### Characterization of the compounds present in the orange essential oil

The chemical composition of the orange essential oil was characterized at the Laboratory of Oils and Fats, Department of Agroindustry, Food and Nutrition (LAN/ESALQ/USP), University of São Paulo, Piracicaba, Brazil. Volatile compounds were identified by headspace gas chromatography–mass spectrometry (HS-GC/MS) using a GCMS-QP2010 Plus system (Shimadzu Corp., Tokyo, Japan) equipped with an Rtx-5MS capillary column. Helium was used as the carrier gas. Compound identification was based on mass spectral comparison with commercial libraries (Wiley 8 and FFNSC 1.3) and confirmation by calculation of linear retention indices using a C7–C30 n-alkane series.

### Feed management and sampling

Coastcross hay was coarsely chopped (DPM – 4 mill, Nogueira, Itapira, São Paulo, Brazil) equipped with a 10-mm pore sieve. The flint corn used herein was processed through a hammer mill (DPM – 4 mill, Nogueira, Itapira, São Paulo, Brazil). Ingredients were individually weighed using a digital scale (0.100 kg) and manually mixed.

The diets were prepared with a pre-mix containing ground corn, soybean meal, ammonium chloride, limestone, and a mineral premix in a horizontal mixer with a capacity of 500 kg (Lucato, Limeira, São Paulo, Brazil). For OEO0, the chopped Coastcross hay was mixed in the feed mixer with the other ingredients. For M25, all ingredients of the OEO0 diet plus sodium monensin (Elanco Animal Health, São Paulo, SP, Brazil) were mixed in the same way as the OEO0. Twenty-five milligrams of monensin (Elanco Animal Health, São Paulo, SP, Brazil) was added per kilogram of diet (DM basis). Monensin was initially mixed with concentrate ingredients by using a mixer with a 500 kg capacity. Thereafter, chopped hay was weighed and added to the concentrate mix to produce the TMR.

For OEO100, OEO500, and OEO1000 diets the orange essential oil was weighed daily on an analytical balance with an accuracy of 0.0001 g (Sartorius BA11OS, Goettingen, Germany) and then added to the pre-mix. Subsequently, chopped hay was weighed and added manually to the concentrate mix to produce the TMR, to obtain a uniform distribution of the OEO. The preparation of OEO100, OEO500, and OEO1000 was carried out every day, throughout the experimental period.

Diets were offered ad libitum as total mixed ration (TMR) once a day at 7:00 h targeting 10% refusals. Samples of the feed offered of approximately 50 g were collected during five consecutive days from the 22nd to the 26th of each period and grouped by period and treatment for subsequent analysis. The waste was weighed daily to calculate the daily quantity offered based on the previous DMI. Samples of the refusals were collected during five consecutive days from the 22nd to the 26th of each period and grouped by period and treatment for subsequent chemical analysis. Samples of diets, refusals and ingredients from each period were sampled and immediately frozen at -18 ˚C.

### Apparent digestibility of nutrients

The intake and the apparent digestibility of nutrients were evaluated over five consecutive days from d 22 through d 26. Intakes were calculated based on DM offered after subtracting DM refused. The total amount of feces was collected to estimate the apparent total digestibility of dietary nutrients. A subsample of 10% of the feces was taken after homogenization, and frozen at -18 °C for later analysis.

### Nitrogen balance

Nitrogen balance was calculated from N in the feed consumed minus the output in feces and urine. As for feces, the urine collection was collected from d 22 through d 26 at 07:00 h. The urine was collected and processed according to Dias Junior et al. (2023a). For pH measurement, pH indicator strips (MQuant Merck, Darmstadt, Germany) were used. The total volume of urine was recorded daily, sampled (10%) and frozen at -18 °C for later analysis.

### Ruminal fluid collection

On d 27, approximately 200 mL of ruminal fluid was manually obtained from each wether via ruminal cannula at 0, 4, 8, 12, 16, 20, and 24 h after feeding. Ruminal pH measurement and ruminal fluid sampling to determine SCFA and ammonia nitrogen in the rumen occurred as described by Dias Junior et al. (2023a).

### Laboratory analyses and calculations

Feed, refusals, and fecal samples were thawed, and dried in a forced-air oven (55 °C for 72 h), and ground through a 1-mm screen of a Wiley-type mill (Marconi, Piracicaba, São Paulo, Brazil) before chemical analysis. Samples were analyzed for DM, organic matter (OM), crude protein (CP) and ether extract according to methods #930.15, #942.05, #990.03 and #920.39) respectively, of the Association of Official Analytical Chemists – AOAC (1990). Neutral detergent fiber (NDF) was determined according to Van Soest et al. (1991). Acid detergent fiber (ADF) was determined in accordance with Goering and Van Soest (1970).

Non-fibrous carbohydrates (NFC) was calculated as: NFC (%) = 100% - (%NDF + % CP + % EE + % MM) according to NRC (2001). Total digestible nutrients (TDN) was calculated using the following equation: TDN (%) = %CP_dig_. + (%EE_dig_. × 2.25) + %NDF_dig_. + NFC_dig_. Metabolizable energy (ME) was estimated using the Cornell Net Carbohydrate and Protein System for sheep (Cannas et al. 2004), which predicts ME supply from feed carbohydrate and protein pools based on ruminal degradation rates and passage. Apparent nutrient digestibility and nitrogen balance were calculated as follows: Apparent digestibility (g/day) = nutrient intake (g/day) - fecal output (g/day). N absorbed (g/day) = N intake - N fecal; N retained (g/day) = N absorbed - N urine; N retained (g/kg N intake) = [N retained (g/day) / N intake (g/day)] × 1000; N retained (g/kg N absorbed) = [N retained (g/day) / N absorbed (g/day)] × 1000.

The SCFA concentration in ruminal fluid was determined according to Ferreira et al. (2016) and ammonia nitrogen was determined according to Broderick and Kang (1980). For statistical analysis, data were transformed to the molar ratio (mM/100 mM, i.e., the ratio between the amount of a given SCFA and the total amount observed). The total pH area under the curve and the area under pH 5.5 were calculated as described by Maulfair et al. (2013).

### Statistical analysis

Power analysis was conducted using the PROC POWER (SAS, 2018) to determine the number of experimental animals and samples based on DMI. The power analysis of the test considered α = 0.05 and power > 0.80. To detect minimal differences in studies that collect biological variables, a test power > 0.80 with α = 0.05 is recommended by Festing and Altman (2002) and Naseri et al. (2022).

Data were analyzed using the MIXED procedure of SAS (2018). All data were subjected to the Shapiro-Wilk test for normality of residuals and removal of outliers. The homogeneity of variances by Levene’s test. The statistical model for repeated measures was: Y = µ + Q_m_ + A_i_(Q_m_) + T_j_ + P_k_ + E_ijk_ + H_l_ + Q_m_ + (T×H)_jl_ + E_ijklm_, where µ = overall mean, Q_m_ = Latin square fixed effect (m = 1 to 2), A_i_(Q_m_ )= animal inside Latin square effect (i = 1 to 10), T_j_ = treatment fixed effect (j = 1 to 5), P_k_ = period random effect (l = 1 to 5), E_ijk_ = random residual error A, H_l_ = fixed effect of hours after feeding, (T×H)_jl_ = fixed effect of interaction between treatment and hours after feeding, and E_ijkl_ = random residual error B. The covariance matrices were tested and defined according to the smallest value obtained for “Akaike’s Information Criterion Corrected” (AICC). The covariance matrix that best fitted the data sets was the compound symmetric (CS). The averages for each treatment were obtained using the LSMEANS command. For the nutrient intake and apparent digestibility, and nitrogen balance, the following model was used: Y = µ + Q_m_ + A_i_(Q_m_) + T_j_ + P_k_ + E_ijk_, where: µ = overall mean, Q_m_ = Latin square fixed effect (m = 1 to 2), A_i_ (Q_m_)= animal inside Latin square effect (i = 1 to 10), T_j_ = treatment fixed effect (j = 1 to 5), P_k_ = period random effect (l = 1 to 5), E_ijk_ = random residual error. The effects of the inclusion levels of OEO in the diets were evaluated using linear (L) and quadratic (Q) orthogonal polynomials. The PROC IML from SAS (2018) was used to obtain the appropriate coefficients for the orthogonal contrasts. To compare the effect of the treatments, two previously defined contrasts were performed: 1 - diets with OEO vs. diet with sodium monensin (OEO vs. M25) and 2 - diet with sodium monensin vs. diet without additive (M25 vs. OEO0). The effects of the periods and the interaction between treatments and periods were defined by the F-test of the analysis of variance (ANOVA). Effects were declared significant when *P* < 0.05.

## Results

### Composition of orange essential oil

The main volatile compounds identified in OEO are shown in Table 2. The analysis showed that D-limonene is the major compound, followed by myrcene and α-pinene.

**Table 2:** Composition of volatile compounds in orange essential oil (*Citrus sinensis*)

Compound^1^	%
D-limonene	95.1
Myrcene	3.8
α-pinene	1.1

^1^Relative amount of identified compounds based on the area of each peak in the chromatogram

### Nutrient intake and apparent digestibility

Nutrient intake was not affected by increasing levels of OEO (*P* > 0.05; Table 3). The treatments with OEO had a higher DM, OM, CP, NDF, NFC and TDN intake than M25 (*P* < 0.05). However, the intake of EE and ADF did not differ in this same contrast. No differences in nutrient intake were observed between M25 and OEO0 (Table 3).

**Table 3:** Effects of orange essential oil (OEO) on intake and apparent nutrient digestibility in sheep

Item^4^	Treatments^1^					SEM^2^	*P*-value^3^			
	OEO0	OEO100	OEO500	OEO1000	M25		L	Q	OEO vs. M25	M25 vs. OEO0
*Intake* (g/day)										
DM	855.9	974.6	866.8	843.3	756.0	52.26	0.25	0.09	0.03	0.19
OM	808.4	925.2	820.8	797.7	717.8	49.37	0.24	0.08	0.03	0.20
CP	135.5	155.1	138.4	131.8	119.4	8.27	0.20	0.09	0.03	0.18
EE	29.2	33.1	28.9	27.2	25.7	1.79	0.36	0.09	0.06	0.18
NDF	140.1	157.7	142.5	139.9	124.4	8.44	0.24	0.14	0.03	0.20
ADF	56.0	60.9	55.4	53.2	50.0	3.32	0.48	0.24	0.11	0.23
NFC	503.6	579.3	510.9	498.8	448.3	31.26	0.26	0.08	0.03	0.22
TDN	761.1	868.5	778.1	760.7	674.5	51.21	0.25	0.14	0.04	0.24
*Digestibility* (g/day)										
DM	849.8	845.2	843.1	851.5	844.5	4.39	0.42	0.81	0.70	0.42
OM	869.7	865.6	863.6	876.3	874.5	7.33	0.16	0.89	0.31	0.50
CP	861.3	858.8	856.9	862.5	868.1	9.94	0.35	0.99	0.27	0.48
EE	698.2	624.9	667.4	666.7	719.0	37.14	0.28	0.22	0.14	0.69
NDF	656.4	655.2	621.0	634.8	595.6	19.42	0.84	0.54	0.08	0.04
ADF	442.7	441.0	407.1	501.7	488.8	25.01	0.06	0.53	0.16	0.19
NFC	979.3	981.5	981.6	975.3	971.6	1.78	< 0.01	0.68	< 0.001	< 0.01
TDN	849.8	845.2	843.1	851.5	844.5	4.39	0.42	0.81	0.70	0.42

^1^OEO0: inclusion of 0 mg/kg DM of orange essential oil; OEO100: inclusion of 100 mg/kg DM of orange essential oil; OEO500: inclusion of 500 mg/kg DM of orange essential oil; OEO1000: inclusion of 1000 mg/kg DM of orange essential oil; M25: inclusion of 25 mg/kg DM of sodium monensin

^2^SEM: Standard error of the means

^3^L: linear effect; Q: quadratic effect; OEO vs. M25: treatments containing orange essential oil vs. treatment with 25 mg/kg DM of sodium monensin (negative control vs. positive control); M25 vs. OEO0: treatment with 25 mg/kg DM of sodium monensin vs. treatments without orange essential oil (positive control vs. negative control)

^4^DM: dry matter; OM: organic matter; CP: crude protein; EE: ether extract; NDF: neutral detergent fiber; ADF: acid detergent fiber; NFC: non-fibrous carbohydrate; TDN: total digestible nutrients

Increasing OEO did not affect the apparent digestibility of DM, OM, CP, EE, NDF, ADF, and TDN (*P* > 0.05; Table 3). However, the apparent digestibility of NFC decreased linearly when OEO was added (*P* < 0.01; Table 3). When OEO diets were compared with M25, apparent digestibility of NFC was greater in OEO-fed animals (*P* < 0.001), while no differences were observed for the remaining nutrients. Compared with OEO0, the M25 resulted in lower apparent digestibility of NDF and NFC (*P* < 0.05), with no effects on other digestibility coefficients.

### N balance

Nitrogen (N) intake was not affected by increasing OEO in the diets (*P* > 0.05; Table 4). However, treatments with OEO showed higher N intake when compared to M25 (*P = 0.03*). Nitrogen excretion in feces and urine, N absorption and N retention were not influenced by the treatments (*P* > 0.05; Table 4).

**Table 4:** Effects of orange essential oil (OEO) on nitrogen balance in sheep

Item^4^	Treatments^1^					SEM^2^	*P*-value ^3^			
	OEO0	OEO100	OEO500	OEO1000	M25		L	Q	OEO vs. M25	M25 vs. OEO0
N intake, g/day	21.7	24.8	22.2	21.1	19.1	1.5	0.20	0.09	0.03	0.18
N fecal, g/day	3.1	3.4	3.2	2.9	2.6	0.1	0.12	0.08	0.23	0.35
N urine, g/day	10.8	12.9	11.2	11.0	10.9	0.9	0.51	0.10	0.42	0.93
N absorbed, g/day	18.5	21.4	18.9	18.2	16.5	1.5	0.27	0.11	0.06	0.27
N retained										
g/day	7.8	9.3	7.7	7.2	5.6	1.5	0.46	0.35	0.11	0.24
g/kg of N intake	338.9	348.9	332.9	305.8	253.5	40.5	0.48	0.84	0.12	0.14
g/kg of N absorbed	394.2	405.4	388.9	355.9	289.6	45.9	0.42	0.85	0.09	0.12

^1^OEO0: inclusion of 0 mg/kg DM of orange essential oil; OEO100: inclusion of 100 mg/kg DM of orange essential oil; OEO500: inclusion of 500 mg/kg DM of orange essential oil; OEO1000: inclusion of 1000 mg/kg DM of orange essential oil; M25: inclusion of 25 mg/kg DM of sodium monensin

^2^SEM: Standard error of the means

^3^L: linear effect; Q: quadratic effect; OEO vs. M25: treatments containing orange essential oil vs. treatment with 25 mg/kg DM of sodium monensin (negative control vs. positive control); M25 vs. OEO0: treatment with 25 mg/kg DM of sodium monensin vs. treatments without orange essential oil (positive control vs. negative control)

^4^N: nitrogen

### Ruminal fermentation characteristics

Increasing OEO did not affect total SCFA concentration, individual SCFA proportions, or the acetate: propionate ratio (*P* > 0.05; Table 5). Overall, ruminal fermentation characteristics were similar between OEO and M25 treatments. When compared with OEO0, the M25 resulted in lower total SCFA concentration (*P* < 0.05), whereas individual SCFA proportions and the acetate: propionate ratio were not affected (Table 5).

**Table 5:** Effects of orange essential oil (OEO) on ruminal fermentation characteristics in sheep

Item^4^	Treatments^1^					SEM^2^	*P*-value ^3^					
	OEO0	OEO100	OEO500	OEO1000	M25		L	Q	OEO vs. M25	M25 vs. OEO0	H	T×H
SCFA total (mM)	60.6	50.1	56.1	50.1	50.4	1.07	0.85	0.06	0.66	0.05	< 0.0001	0.77
SCFA (mM/100 mM)												
Acetate	55.9	55.2	56.3	59.3	54.9	1.80	0.73	0.69	0.36	0.74	< 0.0001	0.26
Propionate	29.6	28.4	27.9	25.4	30.1	2.52	0.54	0.92	0.35	0.88	< 0.0001	0.33
Butyrate	11.0	11.8	11.8	12.3	11.1	1.26	0.62	0.81	0.57	0.96	0.04	0.98
Isobutyrate	0.64	0.73	0.68	0.73	0.74	0.047	0.93	0.20	0.66	0.15	< 0.0001	0.28
Valerate	1.81	2.28	2.15	1.54	2.14	0.304	0.52	0.41	0.67	0.44	0.19	0.96
Isovalerate	1.19	1.37	1.42	1.49	1.36	0.131	0.31	0.68	0.68	0.36	< 0.0001	0.35
A: P	2.23	2.45	2.73	2.80	2.26	0.271	0.16	0.94	0.22	0.93	< 0.0001	0.12
pH	5.81	5.89	5.80	5.93	5.92	0.068	0.56	0.31	0.58	0.27	< 0.0001	0.97
pH area < 5.5 (uni. pH × h/d)	49.6	49.9	51.3	44.8	34.3	9.78	0.47	0.94	0.22	0.28	-	-
pH area total (uni. pH × h/d)	136.9	138.5	136.9	140.4	139.9	1.66	0.58	0.41	0.52	0.22	-	-
Ammonia (mg/dL)	18.9	18.1	19.7	18.9	17.8	0.89	0.24	0.21	0.24	0.33	< 0.0001	0.99

^1^OEO0: inclusion of 0 mg/kg DM of orange essential oil; OEO100: inclusion of 100 mg/kg DM of orange essential oil; OEO500: inclusion of 500 mg/kg DM of orange essential oil; OEO1000: inclusion of 1000 mg/kg DM of orange essential oil; M25: inclusion of 25 mg/kg DM of sodium monensin

^2^SEM: Standard error of the means

^3^L: linear effect; Q: quadratic effect; OEO vs. M25: treatments containing orange essential oil vs. treatment with 25 mg/kg DM of sodium monensin (negative control vs. positive control); M25 vs. OEO0: treatment with 25 mg/kg DM of sodium monensin vs. treatment without orange essential oil (positive control vs. negative control)

^4^SCFA: short-chain fatty acid; A:P: acetate: propionate ratio

The experimental diets did not affect the rumen pH, the area under pH 5.5, the total area under the curve, nor the ammonia nitrogen (*P* > 0.05; Table 5).

## Discussion

### Nutrient intake and apparent digestibility

The similar DMI after increasing OEO in the diet in the current study is consistent with the similarity in the chemical composition of all experimental diets coupled with a lack of effect of OEO on DM digestibility and rumen fermentation characteristics. Literature indicates that the absence of effects on digestibility, ruminal fermentation, and dry matter intake may be related to the capacity of bioactive compounds present in essential oils to be absorbed in the ruminal epithelium, degraded by microbiota, or rapidly pass through the rumen (Cobellis et al. 2016; Torres et al. 2020; Dorantes-Iturbide et al. 2022). These mechanisms may reduce the effective bioavailability of bioactive compounds in the ruminal environment. Currently, the understanding of how compounds present in essential oils are absorbed or metabolized in the rumen is not completely clear, highlighting a knowledge gap that needs to be filled to guide adjustments in supplementation levels in future research exploring essential oils as nutritional additives. However, Maggiolino et al. (2022) highlighted that kids receiving D-limonene via diet (oral administration) had a higher concentration of D-limonene in the meat, indicating a high absorption rate of this compound by the ruminal epithelium.

The higher DMI in wethers fed with OEO compared with a treatment containing M25 is consistent with by the dose of sodium monensin used in the present study. Polizel et al. (2021) reported that the dose of 24 mg/kg DM of sodium monensin reduced the DMI of lambs feed a high-energy diet, which corroborates the results of the present study using a slightly higher dose of 25 mg/kg DM sodium monensin in a diet of similar characteristics (i.e. high-concentrate inclusion). Furthermore, the higher DMI observed in animals supplemented with OEO compared with MON may be attributed to the lower palatability of monensin, as no differences were found in digestibility or ruminal fermentation parameters. Literature suggests that monensin has lower palatability for ruminants compared to essential oils (Baile et al. 1979; Segabinazzi et al. 2011). Essential oils are known to enhance diet palatability, stimulating taste and acceptability (Franz et al. 2009; Mucha and Witkowska 2021), which may explain the increase in DMI observed with essential oil supply.

Increasing OEO in the diet decreased the NFC digestibility. It is reported that D-limonene affects gram-negative bacteria (Gupta et al. 2021), which are responsible for the digestion of NFC in the rumen. Therefore, this response suggests a dose-dependent antimicrobial effect of OEO, resulting in the reduction of the apparent NFC digestibility. In turn, the higher digestibility of the NFC in the treatments with OEO compared to the M25 was consistent with the fact that wether fed OEO had higher DMI (Table 3). There was lower NFC digestibility for M25 compared to OEO0. This effect was unexpected, as similar doses of sodium monensin used in concentrate-based diets did not cause a reduction in NFC digestibility (Muntifering et al. 1981; Gonzalez-Momita et al. 2009). However, it has already been described that sodium monensin can reduce the digestibility of ruminal starch when associated with high-concentrate diets (Muntifering et al. 1981; Spears 1990). This mechanism may explain the lower digestibility of NFC when the wethers were fed 25 mg/kg DM of sodium monensin.

### Nitrogen balance

The higher nitrogen (N) intake observed in sheep fed with OEO compared to M25 is consistent with the increased crude protein (CP) intake in the OEO treatments. However, the lack of significant differences in N excretion via feces and urine, as well as in the amounts of N absorbed and retained, associated by the similarity in CP digestibility among the treatments.

Despite higher N intake with OEO vs. M25, N excretion, absorption, and retention remained similar across treatments, consistent with essential oil studies showing no changes in N utilization efficiency despite variable intake (Busquet et al. 2006; Dias Junior et al., 2023a).

Crude protein digestibility plays a critical role in nitrogen metabolism, as it directly affects fecal and urinary N excretion, along with the amounts of N absorbed and retained (Broderick and Reynal 2009; Mutsvangwa et al. 2016). These findings align with Luo et al. (2015), who reported comparable N retention in sheep fed natural additives despite differences in N intake. Consequently, similar CP digestibility among treatments results in comparable N excretion, absorption, and retention, even when CP intake differs. These findings highlight the central role of CP digestibility in evaluating the effects of feed additives on nitrogen metabolism and their impact on productive efficiency.

### Ruminal fermentation characteristics

In the present study, there were no additional positive nor deleterious effects compromising rumen fermentation in rams fed OEO1000. The absence of effects on ruminal fermentation parameters may be associated with the possible absorption capacity by the ruminal epithelium or degradation of bioactive compounds from OEO in the rumen. Future studies evaluating higher OEO inclusion levels may help identify supplementation thresholds capable of modulating ruminal fermentation, based on the profile of the oil being utilized as suggested by Benchaar et al. (2008).

Despite the latter statements, there was a reduction in total SCFA concentration for the M25 compared to OEO0 which is consistent with the lower NDF and NFC digestibility for the M25. The lower digestibility of NDF when M25 was compared to OEO0 is in accordance with the literature, as reported when monensin is included in the diet (Dellaqua et al. 2024; Bell et al. 2017). Monensin has a selective antimicrobial effect, acting on Gram-positive bacteria, which are responsible for the degradation of the fibrous fraction of the diet (Russel and Strobel 1989; Luo et al. 2024). Conversely, the lower digestibility of NFC for M25 compared to OEO0 may be associated with the lower DMI observed for M25. Although this parameter did not differ, DMI was 13% lower when lambs received the M25 diet, indicating that the NFC intake per gram of DM consumed was lower for M25 compared to OEO0. It is also worth noting that monensin has been associated with a reduction in ruminal starch digestibility (Spears 1990; Teixeira et al., 2020; Dellaqua et al. 2024), which may negatively impact NFC digestibility, as the diets used contained corn as the main source of NFC, which was included in the same proportion in M25 and OEO0.

The rumen pH was not affected by OEO supplementation. The absence of effect on the area under pH 5.5, and the total area under the pH curve indicate that the experimental treatments acted similarly on rumen pH stability. These results suggest that the inclusion of OEO up to the dose of 1000 mg/kg of DM resulted in ruminal pH responses comparable to those observed with monensina, which is supported by the absence of effect on the evaluated ruminal fermentation parameters in the present study. This finding indicates that higher DMI associated with OEO did not compromise ruminal pH stability under the experimental conditions.

Increases in the level of OEO supplementation did not affect the ammonia nitrogen. This result agrees with Samii et al. (2016), who observed no change in ammonia nitrogen when using different D-limonene contents in a cattle diet. Benchaar et al. (2008) and Wallace (2004) pointed out that the effects of essential oils on ammonia nitrogen were directly associated with the type of essential oil, the major compound, the dose, and the diet profile used. Accordingly, the combination of a D-limonene–rich essential oil with a high-concentrate diet may result in limited modulation of ruminal ammonia nitrogen under these conditions.

## Conclusion

Under the conditions of the present study, orange essential oil did not promote meaningful changes in overall nutrient digestibility, nitrogen balance, or ruminal fermentation parameters in sheep. The primary response observed was a reduction in non-fibrous carbohydrate digestibility, particularly at the highest inclusion level (1000 mg/kg DM), suggesting a selective effect of orange essential oil on carbohydrate utilization rather than a broad modulation of ruminal function. These findings indicate that the evaluated doses may be insufficient to induce consistent alterations in ruminal fermentation, while higher inclusion levels or different dietary contexts could lead to distinct responses. Further research focusing on rumen microbial dynamics and bioavailability of D-limonene is warranted to clarify the mechanisms underlying the observed reduction in non-fibrous carbohydrate digestibility and to better define the potential of orange essential oil as a nutritional additive.

## Supplementary Information

Below is the link to the electronic supplementary material.


Supplementary Material 1



Supplementary Material 2


## Data Availability

The datasets generated in the current study are available from the corresponding author on reasonable request.
