# Supplementary material for: Orange (Citrus sinensis) essential oil as feed additive for sheep: nutrient intake and apparent digestibility, nitrogen balance, and rumen fermentation characteristics
Source: Trop Anim Health Prod. 2026 Mar 9;58(2):161. doi: 10.1007/s11250-026-04969-z (PMC12971922; doi:10.1007/s11250-026-04969-z)
Supplement: Supplementary file 1 — Supplementary Material 1 [file 11250_2026_4969_MOESM1_ESM.docx]

June 20^th^, 2025

Dear Editor,

We are submitting for review the manuscript entitled " **Orange** (*Citrus sinensis*) **essential oil as feed additive for sheep: nutrient intake and apparent digestibility, nitrogen balance, and rumen fermentation characteristics**". This article is part of the research of Paulo César Gonzales Dias Junior (first author), developed by him during his Ph.D. in conjunction with the research group coordinated by me.

In this manuscript, we investigate the effects of three levels of orange essential oil as a feed additive. This article presents in an unprecedented way the effects of orange essential oil on ruminal fermentation characteristics as a feed additive for feedlot lambs. Furthermore, the article showed that orange essential oil presented properties like sodium monensin in modulating rumen fermentation. It is possible to point out the great potential of orange essential oil in replacing sodium monensin at a dose of 25 mg/kg DM in high-concentrate diets for lambs. This article allows the identification of new research gaps, which can contribute to the direction of the scientific community in conducting future research.

We confirm that this manuscript has not been published elsewhere and is not being considered by another journal. We also confirm that no known conflicts of interest are associated with this publication.

All authors have approved the manuscript and agreed to its submission to Tropical Animal Health and Production.

Please let us know if there are any pending or possible corrections.

Thank you very much for your kindness and attention.

My best regards.
